# Local mapping of detector response for reliable quantum state estimation

**DOI:** 10.1038/ncomms5332

**Published:** 2014-07-14

**Authors:** Merlin Cooper, Michał Karpiński, Brian J. Smith

**Affiliations:** 1 Clarendon Laboratory, University of Oxford, Parks Road, Oxford OX1 3PU, UK

## Abstract

Improved measurement techniques are central to technological development and foundational scientific exploration. Quantum physics relies on detectors sensitive to non-classical features of systems, enabling precise tests of physical laws and quantum-enhanced technologies including precision measurement and secure communications. Accurate detector response calibration for quantum-scale inputs is key to future research and development in these cognate areas. To address this requirement, quantum detector tomography has been recently introduced. However, this technique becomes increasingly challenging as the complexity of the detector response and input space grow in a number of measurement outcomes and required probe states, leading to further demands on experiments and data analysis. Here we present an experimental implementation of a versatile, alternative characterization technique to address many-outcome quantum detectors that limits the input calibration region and does not involve numerical post processing. To demonstrate the applicability of this approach, the calibrated detector is subsequently used to estimate non-classical photon number states.

## Abstract (web summary)

The successful realization of quantum information protocols relies on characterization of quantum states and measurements. Here, Cooper *et al.* experimentally demonstrate a technique enabling calibration of a detector with a sizeable number of outcomes using a limited amount of resources.

## Graphical abstract

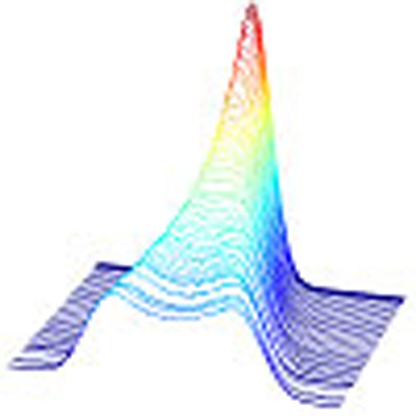

Experimental quantum optics relies on the ability to create, manipulate and measure the state of the light field for applications ranging from fundamental scientific research^1^,^2^,^3^,^4^ to the development of quantum technologies that harness the non-classical behaviour of light^5^,^6^,^7^,^8^. Methods to characterize each step of a quantum experiment are crucial to ensure appropriate evaluation of tests of theoretical predictions and desired device operation. Well-developed techniques for quantum state estimation (QSE)^9^,^10^,^11^ and quantum process tomography^12^,^13^,^14^,^15^ rely on accurate knowledge of the detector response. Only recently has the independent characterization of quantum detectors with few outcomes been experimentally demonstrated by means of quantum detector tomography (QDT)^16^,^17^,^18^,^19^,^20^,^21^,^22^.

Complete characterization of the detector response through QDT becomes increasingly demanding as the number of detector outcomes grows. The primary roadblocks arise from the need to acquire and analyse expanded data sets to extract the mathematical operators associated with each measurement outcome, which becomes intractable with current experimental and computational capacity. This is due to experimental instability over the time required to collect sufficient data, and the size of the numerical inversion problem in terms of the number of distinct measurement outcomes and unavoidable statistical noise that can distort rare detection events^23^.

Here we present an experimental demonstration of an alternative approach to QSE and QDT, known as the fitting of data patterns (FDP)^24^,^25^, which enables calibration of detectors with a sizable number of outcomes and their subsequent use in state estimation. This technique does not extract the complete set of operators that describe the detector, but rather uses the raw measurement outcome distributions for known input states as the detector calibration—negating the need for post processing. The approach limits the input state space to a finite region of interest set by the experimenter independent of the detector behaviour. We apply the FDP method to a balanced homodyne detector (BHD)^9^,^11^, a central resource in a broad class of quantum optical experiments^11^,^26^, which is yet to be independently characterized. The BHD employed here has more than 150 outcomes, which is an order of magnitude more than any quantum detector characterized to date. To demonstrate the FDP method as a tool for complex detector calibration and QSE, we subsequently present QSE of non-classical states of light using the independently calibrated BHD.

## Results

### QSE with a calibrated detector

In quantum theory, the detector response is mathematically represented by its positive operator-valued measure (POVM) comprising a set of positive operators {*π*_*n*_}, with *n*=1, 2,...*N*, labelling the measurement outcomes. The probability for detector outcome *n* given input state *σ*_*ξ*_ is determined by the Born rule


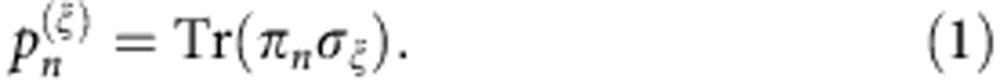


QDT aims to determine the set of measurement operators {*π*_*n*_} by taking the experimentally estimated outcome probabilities 
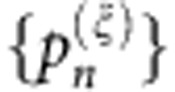
 for a set of known probe states {*σ*_*ξ*_}, and inverting equation (1) (refs 16, 17, 18, 19, 20, 21, 22, 27, 28). To reliably invert equation (1), current QDT techniques depend on registering every measurement outcome of the detector^17^,^19^,^22^,^23^, which requires an increasingly large set of probe states as the number of measurement outcomes and input state space of the detector grow. This leads to greater experimental and computational challenges for reconstruction of the complete POVM set^17^,^19^,^22^,^23^. Although recent work has shown that it may be feasible to address part of the POVM in a truncated input space by artificially limiting the number of detector outcomes^20^, this relies on the same techniques as standard QDT. Having determined the POVM set by QDT, to reconstruct an unknown quantum state one must employ the reconstructed measurement operators within a separate QSE algorithm, for example, maximum-likelihood (ML) estimation^29^,^30^,^31^. Thus, two separate algorithms (and hence two optimization procedures) are employed to perform QSE with calibrated measurements following this approach.

**Figure 1:**
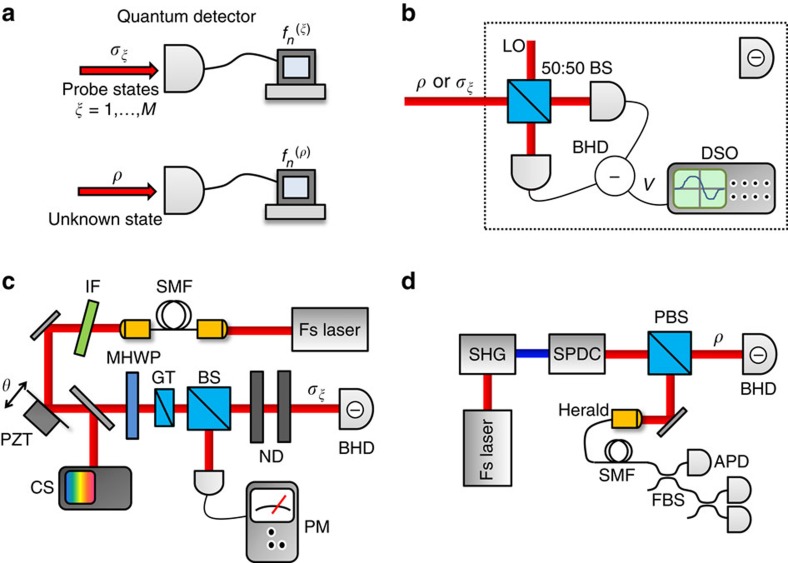
FDP overview and experimental schematic. (**a**) Conceptual and (**b**–**d**) experimental diagrams of the FDP procedure. (**a**) Top: multiple copies of known probe quantum states {*σ*_*ξ*_} are separately measured by the unknown detector to form the data patterns 
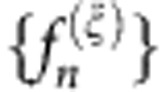
. Bottom: multiple copies of the unknown quantum state *ρ* to be estimated are measured giving the frequency distribution 
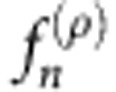
. (**b**) Balanced homodyne detection: probe states {*σ*_*ξ*_} or unknown states *ρ* are combined with the local oscillator (LO) on a 50:50 beam splitter (50:50 BS). The output modes are directed to a pair of photodiodes whose photocurrents are subtracted, with the resulting difference current converted to a voltage measured with a digitizing oscilloscope (DSO)^39^. This yields a set of voltages {*V*} for each state from which the frequency distributions 
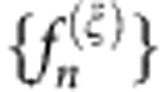
 and 
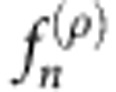
 are determined. (**c**) Probe state preparation: the output of a pulsed Ti:Sapphire laser oscillator is spatially and spectrally filtered with a single-mode fibre (SMF) and interference filter (IF). A motorized half-wave plate (MHWP) and Glan–Taylor polarizer (GT) followed by neutral density (ND) filters control the probe state amplitude, while a piezoelectric transducer (PZT) averages the phase. A calibrated power meter (PM) and compact spectrometer (CS) monitor the optical power and wavelength, respectively, to determine the probe state amplitude. (**d**) Fock state preparation: the Ti:Sapphire laser output is frequency doubled (SHG) to pump a spontaneous parametric downconversion source (SPDC). A spatially multiplexed detector comprising a fibre beam splitter (FBS) network and three avalanche photodiodes (APDs) enables heralding of multi-photon Fock states *ρ*.

The FDP approach begins by recording the detector response to a set of *M* known input probe states {*σ*_*ξ*_} that span the input region of interest. Here *σ*_*ξ*_ is the density operator for the state labelled by *ξ*=1, 2,...*M*. Multiple copies of each probe state are sent to the detector and the frequency distribution 
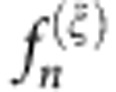
 of measurement outcomes, labelled by *n*, associated with input state *σ*_*ξ*_, is determined as depicted in Fig. 1a. This procedure is similar to mapping the impulse response function of a linear optical system. The data pattern set 
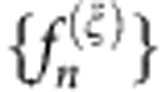
 constitutes the detector calibration within the field of view defined by the probe states. As with QDT, the FDP method relies on the ability to reliably produce a sufficient set of well-known probe states to characterize the detector response^24^,^25^. However, unlike QDT, the data patterns are obtained directly from the measurement outcomes, requiring no additional analysis.

To estimate an unknown quantum state with density operator *ρ* using the FDP approach, many identically prepared copies of the state are measured with the calibrated detector. The frequency distribution of measurement outcomes for the unknown state 
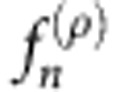
 is determined from the acquired data. The unknown state density operator is estimated as a weighted sum of the probe states


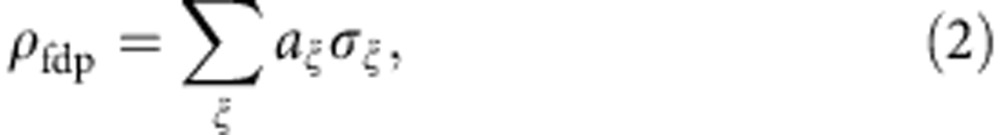


where the real-valued expansion coefficients {*a*_*ξ*_} are found by minimizing the functional


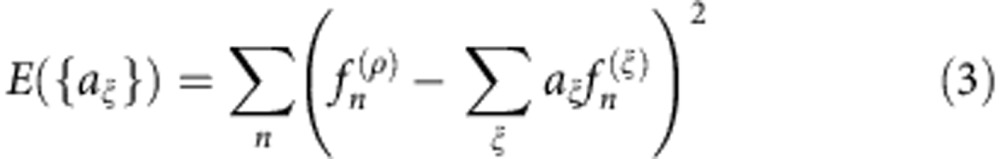


subject to constraints ∑_*ξ*_*a*_*ξ*_=1 and ∑_*ξ*_*a*_*ξ*_*σ*_*ξ*_≥0, which ensure that the reconstructed operator corresponds to a physical state. The FDP algorithm can be formulated as a semi-definite convex program, which can be solved using standard software tools (see Methods). The only assumptions are that the unknown state lies within the calibration field of view defined by the probe states and can be well-represented as a weighted sum of the probe states, equation (2). Note that in the data pattern calibration and state estimation by FDP, the detector POVM {*π*_*n*_} is never revealed, hence the mathematical description of the measurements remains unknown to the experimentalist.

The physical origin for the FDP state estimation approach, encapsulated in the functional of equation (3), can be understood by examining the Born rule, equation (1), applied to the probe states and estimated state. Minimization of the functional in equation (3) aims to reduce the difference between the probability distribution for the unknown state, 
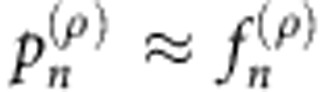
, and that calculated from the estimated state 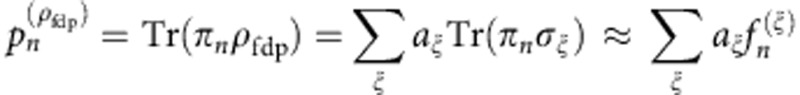, where probabilities are approximated by their corresponding measured frequencies.

The FDP method uses an operational characterization of the detector response, encapsulated in the data pattern set 
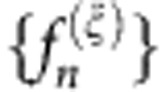
, to perform reliable QSE by minimizing only one functional, equation (3) (refs 24, 25). The detector calibration consists only of the directly obtained measurement outcome distributions and thus does not require further numerical analysis. The alternative approach of performing QDT followed by QSE entails two separate optimization procedures, each performed subject to separate constraints and, in the case of QDT, often regularization^17^,^23^,^28^. This two-step approach to QSE with an unknown detector is not only more resource intensive, owing to the two separate numerical optimizations, but also more likely to suffer from artefacts introduced from one or both of the separate optimization procedures^32^. Although ancilla-assisted detector characterization techniques might offer simpler post processing than standard QDT^27^,^33^, they are limited by the available brightness of the requisite non-classical state^33^. Furthermore, as in the case of standard QDT, a second optimization step to perform QSE using the reconstructed POVM elements is necessary.

### Detector calibration by determination of data pattern set

To experimentally demonstrate the data pattern calibration and its subsequent application to state reconstruction for a detector with a large number of outcomes, we examine a BHD. This vital resource for continuous-variable quantum technologies is typically assumed to have an output that is proportional to the electric field quadrature of a well-defined spatial-temporal optical field mode^9^,^11^. Indeed, balanced homodyne detection was used in the first quantum state and continuous-variable quantum process estimation experiments^14^,^34^. Optical interference of a reference beam called the local oscillator with the unknown signal field to be examined defines the detection mode, as presented in Fig. 1b. The balanced scheme suppresses the local oscillator technical noise and shot noise, allowing ultra-sensitive sampling of the signal.

The BHD is characterized by a set of probe states comprising 48 phase-averaged coherent states with varying amplitudes. The probe states are generated deterministically by attenuating and phase randomizing a laser beam, as shown in Fig. 1c. The use of phase-averaged probes to characterize the detector implies that the calibration field of view enables access to the photon number statistics of the unknown quantum states, which in the case of phase-invariant states constitutes complete QSE.

**Figure 2:**
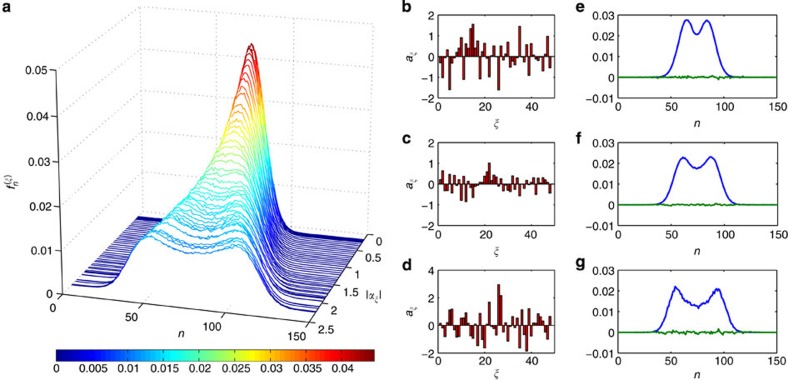
Data pattern set and fitting of data patterns. (**a**) Data patterns 
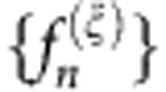
 obtained for the set of 48 phase-averaged coherent state probes {*σ*_*ξ*_} with |*α*_*ξ*_| ranging from 0.17 to 2.24. (**b**–**d**) Optimal coefficients {*a*_*ξ*_} minimizing the functional defined in equation (3) for the one-, two- and three-photon Fock states, respectively. (**e**–**g**) Measured frequency distributions 
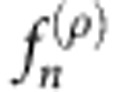
 (blue curves) and residuals between fitted frequency distributions and measured frequency distributions 
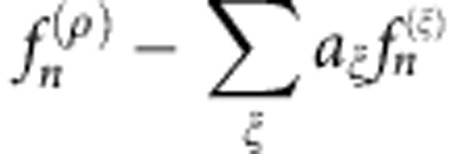
 (green curves) for the one-, two- and three-photon Fock states, respectively, where index *ξ* is in the order of increasing probe state amplitude |*α*_*ξ*_|. The latter gives a measure of the deviation between the measured and predicted frequency distributions, which is found to be within the statistical noise due to the finite number of measurements.

For each probe state *σ*_*ξ*_, an ensemble of *K*=10^6^ optical pulses is measured sequentially by the BHD, yielding a set of voltages 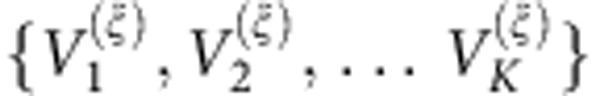 recorded by a fast oscilloscope. In the experiment, the probe state amplitudes |*α*_*ξ*_| range from 0.17 to 2.24 in approximately even steps of 0.043. The frequency distribution 
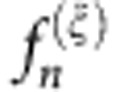
 of measurement outcomes *n* for state *σ*_*ξ*_ is formed by binning the voltage samples {*V*′} after rescaling (see Methods). Each frequency distribution consists of 151 bins, implying as many detector outcomes, which is more than an order of magnitude greater than any QDT experiment to date. The procedure is repeated for each probe state *σ*_*ξ*_ to give the detector data pattern set 
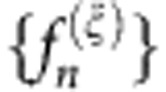
, as shown in Fig. 2a, constituting the detector calibration. Note that no physical interpretation about the nature of the measurement outcomes is necessary in the FDP method^24^,^25^.

### Estimation of non-classical states using calibrated detector

Several non-classical photon number (Fock) states^35^,^36^,^37^,^38^ are examined using the calibrated detector. The density matrices *ρ* are estimated by fitting the probe state data patterns 
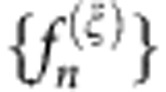
 to the frequency distribution of measurement outcomes 
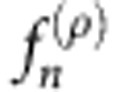
 for heralded one-, two- and three-photon Fock states according to equation (3). The Fock states are generated by pulsed spontaneous parametric downconversion (see Methods) and measured by the BHD. Frequency distributions 
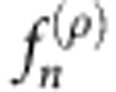
 for each Fock state are obtained in the same manner as for the probe states, shown in Fig. 2. The functional defined in equation (3) is minimized for each state separately to determine the optimal set of coefficients {*a*_*ξ*_}, shown in Fig. 2b–d.

**Figure 3:**
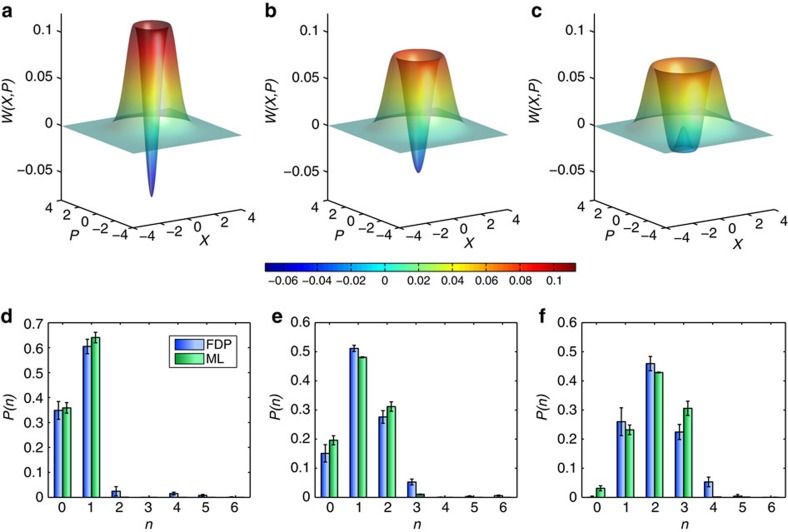
Non-classical state reconstruction. Reconstructed Wigner functions *W*(*X*,*P*) from FDP method (**a**–**c**) and photon number statistics *P*(*n*) (**d**–**f**) from FDP (blue bars) and ML (green bars) methods for the (**a**,**d**) one-, (**b**,**e**) two- and (**c**,**f**) three-photon Fock states. The reconstructed Wigner functions exhibit negative values, thus indicating the non-classical nature of the reconstructed states. Error bars indicate one-*σ* confidence intervals and include both systematic and statistical sources of error for both the FDP and ML state reconstructions (see Methods).

The reconstructed Wigner functions *W*(*X*,*P*) and photon number statistics *P*(*n*)=〈*n*|*ρ*_fdp_|*n*〉 for the generated Fock states are shown in Fig. 3. To compare with the commonly used ML method for QSE, the same homodyne data that yield the frequency distributions 
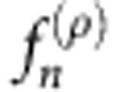
 are used in a ML QSE algorithm^11^. Here, a POVM of the form {*π*_*X*_}={|*X*〉〈*X*|} and phase-invariant states are assumed, where |*X*〉 is the quadrature eigenstate with eigenvalue *X* (see Methods). The photon number distributions reconstructed with the ML estimation are shown in Fig. 3. The fidelity 
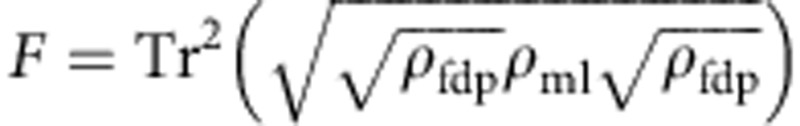
 between density matrices *ρ*_fdp_ and *ρ*_ml_ estimated by the FDP and ML methods for each Fock state is calculated, yielding fidelities *F*_1_=0.96±0.02, *F*_2_=0.98±0.02 and *F*_3_=0.92±0.02 for the one-, two- and three-photon states, respectively. This agreement between FDP and ML approaches indicates that the assumed quadrature POVM used in the ML estimation is accurate within the field of view experimentally examined by the probe state calibration set and experimental uncertainties. It also provides a self-consistency check between the two methods, thus demonstrating the applicability of FDP method to characterization of many-outcome detectors and QSE.

## Discussion

The ability to accurately calibrate the response of detectors is essential to the progress of quantum technologies. As quantum devices grow in size and complexity, so too will the measurement devices required for their operation and diagnosis. This necessitates the development of techniques to characterize increasingly elaborate detector responses. Standard QDT gives a complete global view of the detector and thus constitutes its characterization to all possible input states^16^,^17^,^19^,^22^,^23^. Such calibration requires eliciting all measurement outcomes by probing the detector in large regions of input space that potentially have no overlap with states of interest to the experimenter. This leads to significant overheads in terms of experimental effort associated with preparing probe states and acquiring large data sets, and computational resources required to perform the numerical inversion of the data to extract the POVM set. Recent work has indicated that reconstruction of a restricted POVM set in a finite range may be possible^20^, but further investigation on the probe state requirements and generalization to arbitrary detectors is needed. Furthermore, QSE with a well-calibrated detector using QDT requires an additional numerical inversion step that adds to the computational tasks for state estimation.

The data patterns approach to detector characterization and QSE represents a key conceptual shift in both detector calibration and state estimation. This approach provides a direct characterization of the detector response in a finite region of input state space in which we anticipate unknown states to exist. There is no numerical inversion associated with the detector characterization. The calibration consists only of the statistics of measurement outcomes, the data patterns, and in no time is the formal mathematical description of the detector, namely its POVM, revealed. Restricting the calibration region can greatly decrease the experimental and computational challenges, at the cost of limiting the domain over which the detector is characterized. The single-step data processing for state estimation enables increased inversion speed.

Here we have presented an experimental demonstration of a novel method for quantum detector characterization and QSE by the FDP. This approach enables calibration of many-outcome detectors through direct, local measurement of the characteristic detector response to a set of probe states. The experimental calibration of a BHD and its subsequent use in reconstruction of non-classical Fock states presented here not only demonstrates successful implementation of this new detector characterization approach, but also goes an order of magnitude beyond any quantum detector characterization previously demonstrated. The FDP approach is easily adapted to a variety of measurement devices and the experimental implementation presented shows its viability for detectors with complex response. We anticipate that this approach to detector calibration and QSE will become a standard method to characterize measurement response in a local region of input space, adding to and complementing the QDT followed by QSE approach.

## Methods

### Balanced homodyne measurement

A time-domain BHD with a bandwidth of 80 MHz and signal-to-noise ratio of 14.5 dB is used to perform measurements of the probe and signal fields^39^. For each incident optical pulse, the BHD generates a voltage pulse. The BHD voltage *V* is digitized by a computer-controlled digital storage oscilloscope. Drifts in the detector balance and gain are compensated by rescaling the measured voltage according to *V*′=*AV*−*B*. Rescaling parameters *A* and *B* are determined by acquiring pulses when the BHD input is blocked. Voltage samples with the input blocked are constrained to satisfy 〈*V*′〉=*C*_1_ and var(*V*′)=〈*V*′^2^〉−〈*V*′〉^2^=*C*_2_, where *C*_1_ and *C*_2_ are constants, leading to the relations 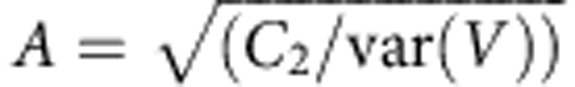 and *B*=*A*〈*V*〉−*C*_1_. The local oscillator repetition rate is 80 MHz, whereas the probe state repetition rate is ∼4 MHz. This enables continuous compensation for any drift^39^. In the case of the ML state reconstruction, where a POVM is assumed, *C*_1_=0 and *C*_2_=1/2 is used, such that *V*′ corresponds to a quadrature eigenvalue *X* for the case of a perfect BHD.

### Probe state preparation

The phase-averaged coherent state probes are derived from a mode-locked Ti:Sapphire oscillator (Spectra-Physics Tsunami) operating at a central wavelength of 830 nm, full-width half-maximum bandwidth of 10 nm and repetition rate of 80 MHz, Fig. 1c. The beam is initially spatially filtered using a single-mode fibre and spectrally filtered using an interference filter (Semrock LL01-830-12.5), to match the spatial-spectral mode of local oscillator. A computer-controlled motorized half-wave plate followed by a Glan–Taylor polarizer and calibrated neutral density filters enables precise control of the probe state amplitude. Phase averaging is achieved by driving a piezoelectric translator on which one of the interferometer mirrors is mounted. The effective probe state amplitude |*α*_*ξ*_| registered by the BHD is given by


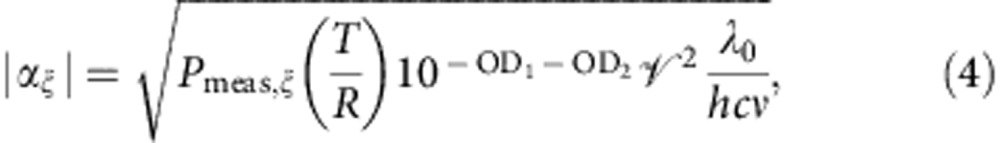


where *P*_meas,*ξ*_ is the average power measured on the calibrated power meter (NIST-traceable Coherent FieldMaxII-TO power meter), *R*(*T*) is the reflectivity (transmissivity) of the beam splitter, OD_1(2)_ is the optical density of neutral density filter 1(2), *ν* is the laser repetition rate, *λ*_0_ is the laser central wavelength and 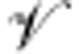 is the interference visibility between the local oscillator mode and probe state mode. The interference visibility is measured by removing the neutral density filters and directing one output of the 50:50 beam splitter used for balanced homodyne detection using a flip mirror to a fast photodiode that records the classical interference pattern as the phase is modulated. A calibrated spectrometer (Thorlabs CCS175) measures the central wavelength *λ*_0_. The probe state spectrum has a central value *λ*_0_=827.6 nm. The laser repetition rate *ν* is measured using a high-precision frequency counter. The repetition rate is decreased by a pulse picker (APE) and was measured to be 3.997 MHz for the duration of the probe state measurement.

### Fock state preparation

A two-mode squeezed vacuum state of the form 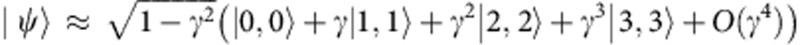, where *γ* is the squeezing parameter and |*n*,*m*〉 is a two-mode state with *n* (*m*) photons in the signal (trigger) mode, is generated by type II pulsed spontaneous parametric downconversion in a bulk potassium di-hydrogen phosphate crystal^40^. The signal and trigger modes are separated by a polarizing beam splitter. A spatially multiplexed detector comprising a fibre beam splitter network and three avalanche photodiodes (Perkin-Elmer SPCM-AQ4C) enables heralding of one-, two- and three-photon Fock states conditioned on registering one, two and three ‘clicks’, respectively, from the multiplexed detector^38^. The heralded Fock state in the signal mode is combined with the local oscillator for performing balanced homodyne detection.

### Reconstruction and uncertainty estimation

Minimization of functional equation (3) subject to constraints ∑_*ξ*_*a*_*ξ*_=1 and ∑_*ξ*_*a*_*ξ*_*σ*_*ξ*_≥0 can be formulated as a semi-definite convex programme. We used the YALMIP^41^ toolbox for MATLAB with the solver SDPT3 (ref. 42) to perform the minimization of equation (3).

The uncertainties in the state fidelities between ML and FDP approaches are estimated by taking into account both statistical and systematic sources of error. Statistical errors arise due to the finite number of measurement events used to generate data patterns and are given by 
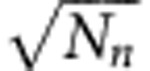
 for each bin *n* with population *N*_*n*_ in the histograms, forming measurement outcome frequency distributions 
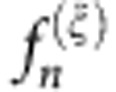
 and 
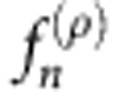
. The bin width is chosen such that each bin is sufficiently narrow to adequately sample the underlying probability distribution^24^. For the FDP state reconstructions, there are also errors originating from the estimates of *P*_meas_ and 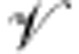 used to determine |*α*_*ξ*_| for each probe state. Uncertainties of 5% in *P*_meas_ and 1% in 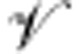 are estimated, which give a total error for each |*α*_*ξ*_| of 2.7%. For the ML state reconstructions, the primary source of error is the uncertainty in detector efficiency *η*_bhd_. This must be independently determined and explicitly taken into account in the ML estimation to compare with the FDP method, which automatically incorporates the detector efficiency. Monte Carlo simulation enables evaluation of the uncertainties for the estimated state density matrix elements and hence also the fidelities when comparing the FDP approach with the results from ML reconstruction. The Monte Carlo simulation takes into account statistical errors in the data patterns and state frequency distributions (as described above), as well as the uncertainty in the probe state amplitudes |*α*_*ξ*_|, which impacts the probe state density matrices {*σ*_*ξ*_} and hence the estimated state *ρ*_fdp_, equation (2).

## Author contributions

M.C. and B.J.S. conceived the project. M.C. designed and performed the experiment. M.C. and M.K. performed modelling and data analysis, including code to implement the algorithm. All authors contributed equally to writing the manuscript.

## Additional information

**How to cite this article:** Cooper, M. *et al.* Local mapping of detector response for reliable quantum state estimation. *Nat. Commun.* 5:4332 doi: 10.1038/ncomms5332 (2014).
